# Causal relationships between genetically determined metabolites and human intelligence: a Mendelian randomization study

**DOI:** 10.1186/s13041-021-00743-4

**Published:** 2021-02-09

**Authors:** Jian Yang, Binbin Zhao, Li Qian, Fengjie Gao, Yanjuan Fan, Xiaoyan He, Qingyan Ma, Lihong Yang, Bin Yan, Wei Wang, Xiancang Ma

**Affiliations:** 1 Clinical Research Center, The First Affiliated Hospital of Xi’an Jiaotong University, Xi’an, China; 2 Department of Psychiatry, the First Affiliated Hospital of Xi’an Jiaotong University, No. 277 Yanta West Road, Xi’an, 700061 China; 3 Center for Brain Science, The First Affiliated Hospital of Xi’an Jiaotong University, Xi’an, China

**Keywords:** Genetically determined metabolite, Human intelligence, Mendelian randomization, Metabolic pathway, 5-Oxoproline

## Abstract

Intelligence predicts important life and health outcomes, but the biological mechanisms underlying differences in intelligence are not yet understood. The use of genetically determined metabotypes (GDMs) to understand the role of genetic and environmental factors, and their interactions, in human complex traits has been recently proposed. However, this strategy has not been applied to human intelligence. Here we implemented a two-sample Mendelian randomization (MR) analysis using GDMs to assess the causal relationships between genetically determined metabolites and human intelligence. The standard inverse-variance weighted (IVW) method was used for the primary MR analysis and three additional MR methods (MR-Egger, weighted median, and MR-PRESSO) were used for sensitivity analyses. Using 25 genetic variants as instrumental variables (IVs), our study found that 5-oxoproline was associated with better performance in human intelligence tests (P_IVW_ = 9.25 × 10^–5^). The causal relationship was robust when sensitivity analyses were applied (P_MR-Egger_ = 0.0001, P_Weighted median_ = 6.29 × 10^–6^, P_MR-PRESSO_ = 0.0007), and repeated analysis yielded consistent result (P_IVW_ = 0.0087). Similarly, also dihomo-linoleate (20:2n6) and p-acetamidophenylglucuronide showed robust association with intelligence. Our study provides novel insight by integrating genomics and metabolomics to estimate causal effects of genetically determined metabolites on human intelligence, which help to understanding of the biological mechanisms related to human intelligence.

## Introduction

Intelligence affects all aspects of human life [1]. During the school years, some individuals show higher intelligence, attain better marks in exams, and have better prospects for further education [2, 3]. In the workplace, intelligence influences performance, efficiency, the ability to cope with difficulties, and career achievements [4]. Intelligence is also a predictor of higher quality of life and better health outcomes [5, 6]. Revealing the biological bases of individual differences in human intelligence has become a central and enduring aim of psychological and brain sciences. During the past decade, advances in genetic research have greatly promoted our understanding of intelligence [7–10]. However, further insight on its biological basis is needed.

Understanding the role of genetic characteristics and their interaction with environmental factors is the key to reveal the biological mechanisms underlying differences in human intelligence [11]. Currently, omics technologies (such as genomics, metabolomics, etc.) are widely used to provide a comprehensive characterization at the molecular level of the human body as a biological system. These approaches have successfully identified a number of informative biomarkers and greatly advanced our knowledge of the molecular mechanisms responsible for many traits. However, most omics studies focus only on a single layer, and therefore fail to capture information across multiple omics assays [12]. Recently, researchers have linked metabolomics traits to genomic information through genome-wide association studies (GWAS) on non-targeted metabolic profiling [13–15]. A large database of genetically determined metabotypes (GDMs) has been thus established to provide comprehensive insights of how genetic variation influences metabolism [16]. The established GDMs provide important intermediates to reveal the role of the interactions between genetics and metabolic traits in determining differences in human intelligence.

Mendelian randomization (MR) is a novel genetic epidemiology study design using genetic variants as instrumental variables (IVs) to investigate whether a modifiable exposure is causally related to a medically relevant disease risk [17]. The fundamental assumption utilized in the MR framework is that if genetic variants essentially affect the biological effects of a modifiable exposure, they should be also related to the exposure-related disease risk. Exploiting the fact that inherent genetic variants are not generally susceptible to environmental variables, the MR design can avoid the potential confounding factors that are common in conventional observational studies [18]. In recent years, the explosion in the number of published GWAS summary data has increased the popularity of MR approaches (and in particular of two-sample MR analysis) as tools to infer the causality of risk factors on complex health outcomes [19–21]. In this study, using GDMs and the results of GWAS on intelligence, we implement two-sample MR analysis to: (1) assess the causal effects of genetically determined metabolites on human intelligence; (2) investigate the genetic basis that may play a central role in determining the variation of the related metabolites and the differences in human intelligence; (3) identify potential metabolic pathways involved in the biological processes related to intelligence.

## Methods

### GWAS scans with metabolomics traits

Shin et al*.* reported the most comprehensive exploration of genetic influences on human metabolism so far, by performing a GWAS of non-targeted metabolomics on 7824 healthy adults. [16]. Metabolic profiling was carried out on fasting serum using high-performance liquid chromatography and gas chromatography separation coupled with tandem mass spectrometry. After quality control, 486 metabolites were retained for genetic analysis, among which 309 were chemically identified and could be further assigned to 8 metabolic groups (amino acids, carbohydrates, cofactors and vitamins, energy, lipids, nucleotides, peptides, and xenobiotics), while the other 177 were classified as ‘unknown’. The final genome-wide association analyses were carried out on approximately 2.1 million single nucleotide polymorphisms (SNPs). Full summary statistics for the 486 metabolites can be found at the Metabolomics GWAS Server (http://metabolomics.helmholtz-muenchen.de/gwas/).

### IVs for the 486 metabolites

The foundational principle of MR relies on the existence of valid IVs. A genetic variant is a valid IV if it is (i) significantly associated with the exposure, (ii) independent of confounders, and (iii) associated with the outcome only through the exposure [22]. To identify valid IVs, we first selected the SNPs with significance P < 1 × 10^−5^, so as to account for a proportion as large as possible of the variance explained for the corresponding metabolite. We next performed a clumping procedure (linkage disequilibrium threshold of r^2^ < 0.1 within a 500-kb window) to select the independent SNPs using the PLINK software (v1.9). To avoid the negative impact of weak IVs, we further used the proportion of variation explained by each IV (*R*^2^) and the *F* statistics to select SNPs strong enough to be valid IVs. Typically, an *F* statistic > 10 is considered sufficient for MR analysis [23].

### GWAS summary data on intelligence

GWAS summary statistics for intelligence were obtained from the study by Savage et al*.* [10]. Briefly, these authors performed a large GWAS meta-analysis of 269,867 individuals from 14 cohorts of European ancestry. Intelligence was assessed using different neurocognitive tests and the general factor of intelligence (Spearman’s *g*). Although differences in assessment methods might reduce the power to detect associations in meta-analyses, this approach can at the same time reduce type I errors by removing measurement errors, and therefore identify SNPs with robust associations to the common latent factor underlying intelligence across different methods. Stringent quality control procedures were applied to the summary statistics for each cohort. Association analysis was conducted controlling for covariates of age, sex, genotyping array, socioeconomic status for specific cohort, and twenty European-based ancestry principal components. Finally, a total of 9,295,118 SNPs were included in the meta-analysis.

### Statistical analysis

Primary two-sample MR analyses were performed using the standard inverse-variance weighted (IVW) method. The IVW method provides a consistent estimate of causal effects by combining the ratio estimates of each variant in a fixed-effect meta-analysis model [23]. The P-value was calculated with a standard normal cumulative distribution function on the ratio of the combined causal effect and its standard error. The significance threshold to declare a causal relationship for the IVW-based MR estimate was set, using Bonferroni correction, at P < 1.03 × 10^–4^ (= 0.05/486). Associations with P < 0.05, but not reaching the Bonferroni-corrected threshold, were reported as suggestive of association.

The IVW method provides an unbiased estimate under the assumption that all genetic variants are valid IVs. However, this assumption is easily violated, leading to inaccurate estimates, when horizontal pleiotropy occurs (some variants act on the outcome via a different intermediary) [24]. To avoid the effects of widespread horizontal pleiotropy in MR, we further performed sensitivity analyses using three additional MR methods: the MR-Egger method, which provides a consistent causal effect estimate, even when all genetic variants violate the assumptions defining valid IVs, under a weaker assumption (known as the InSIDE [instrument strength independent of direct effect] assumption) [24]; the weighted median method, which introduces a weighted median estimator and provides a more precise estimate than MR-Egger regression without the InSIDE assumption [25]; and the MR-PRESSO method, a newly developed approach which can identify and correct for horizontal pleiotropic outliers in MR [26]. We further used the MR-PRESSO global test as well as the intercept of the MR-Egger regression to test for pleiotropy, and we also evaluated heterogeneity with the *I*^2^ and the Cochran Q test. Typically, *I*^2^ > 25% or Cochran Q-derived *P* < 0.05 were used as indicators of possible horizontal pleiotropy. Analyses were carried out using the packages *MendelianRandomization* and *MR-PRESSO* in R (version 3.6.1).

### Replication

We next used GWAS datasets of four other related outcomes to replicate the findings of our MR estimates. The first dataset was obtained from another GWAS of intelligence with 248,482 samples from the UK Biobank [27]. Summary statistics of cognitive performance (n = 257,828) and educational attainment (n = 766,345) were obtained from the study of Lee et al. [28]. Genetic associations with income (n = 286,301) were extracted from the large publicly available Lothian Birth Cohorts of 1921 and 1936 data-sharing resource [29]. Notably, the Davies et al*.* reported another GWAS for intelligence with a larger sample size, but the summary data for full dataset is not available due to data permissions [30].

### Metabolic pathway analysis

Metabolic pathway analysis was carried out using the web-based tool suite MetaboAnalyst 4.0 (https://www.metaboanalyst.ca/) [31]. For this analysis, we extracted all metabolites showing suggestive associations in the IVW estimates (P_IVW_ < 0.05). Two libraries of metabolic pathways or metabolite sets were selected for enrichment analysis, namely the Small Molecule Pathway Database (SMPDB, http://www.smpdb.ca) [32] and the Kyoto Encyclopedia of Genes and Genomes (KEGG, https://www.kegg.jp/) database [33]. P-values < 0.05 were considered statistically significant.

## Results

### Causal effects of the metabolites on intelligence

**Fig. 1:**
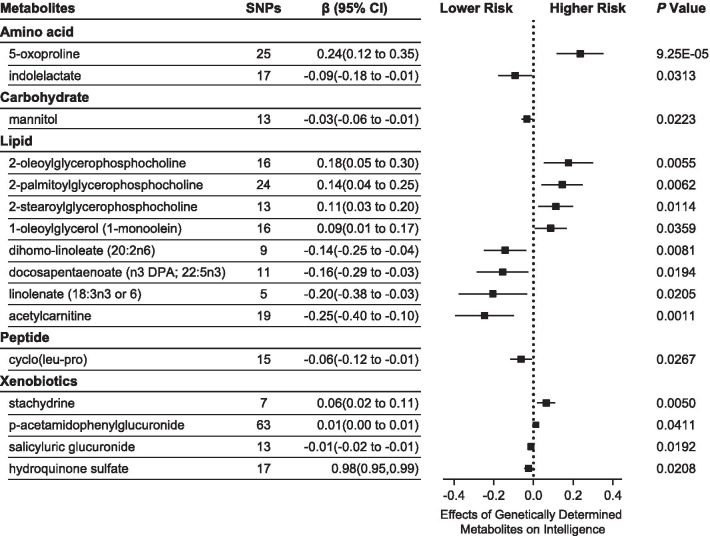
Mendelian randomization associations of genetically determined metabolites on intelligence

We selected 3–675 independent genetic variants as IVs for each of the 486 metabolites (Additional file 4: Table S1). On average, the IVs explained 4.7% (range 0.8–83.5%) of the variance of their respective metabolic traits. The minimum F statistic used to evaluate the strength of these IVs was 20.33. Using these IVs, IVW identified 16 known metabolites and 16 unknown metabolites that might have causal effects on human intelligence (Fig. 1, Additional file 4: Table S2). Among the 16 known metabolic traits, 5-oxoproline was significantly associated with intelligence after Bonferroni correction (P_IVW_ = 9.25 × 10^–5^). Using 25 SNPs as proxy, we observed a 0.24 increase in the score of the Spearman’s *g* test for an increase of one standard deviation (SD) in the level of 5-oxoproline (β = 2.10; 95% Confidence interval [CI] 0.12 to 0.35). We also found 15 other metabolites to be suggestive for association, including indolelactate (β = − 0.09; 95% CI − 0.81 to − 0.01, P_IVW_ = 0.0313), mannitol (β = − 0.03; 95% CI − 0.06 to − 0.01, P_IVW_ = 0.0223), and 2-oleoylglycerophosphocholine (β = 0.18; 95% CI 0.05 to 0.30, P_IVW_ = 0.0055).

### Sensitivity analysis

**Fig. 2:**
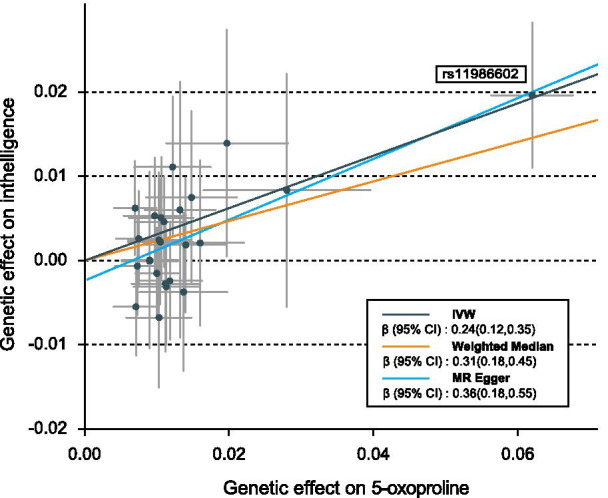
Genetic associations between 5-oxoproline and intelligence

**Fig. 3:**
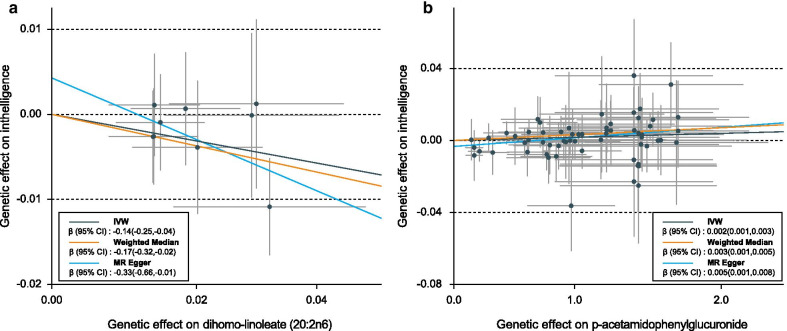
Genetic associations of two suggestive metabolites with intelligence. **a** Dihomo-linoleate (20:2n6); **b** p-acetamidophenylglucuronide

Table 1 shows the results of the sensitivity analyses for the 16 IVW-identified known metabolites. The causal relationship between 5-oxoproline and intelligence was robust when additional MR methods were applied (P_MR-Egger_ = 0.0001, P_Weighted median_ = 6.29 × 10^–6^, P_MR-PRESSO_ = 0.0007), and no horizontal pleiotropy was observed (P_Intercept_ = 0.09, P_Global test_ = 0.06, *I*^2^ = 25%, P_Heterogeneity_ = 0.13). Two other metabolites also showed robust associations with intelligence, namely dihomo-linoleate (20:2n6) (P_MR-Egger_ = 0.0494, P_Weighted median_ = 0.0236, P_MR-PRESSO_ = 0.0293, P_Global test_ = 0.16) and p-acetamidophenylglucuronide (P_MR-Egger_ = 0.0075, P_Weighted median_ = 0.0060, P_MR-PRESSO_ = 0.0454, P_Global test_ = 0.0611), and there were no evidence of horizontal pleiotropy (P_Intercept_ = 0.24, P_Global test_ = 0.17, *I*^2^ = 0%, P_Heterogeneity_ = 0.96 for dihomo-linoleate (20:2n6) and P_Intercept_ =0.06, P_Global test_ = 0.06, *I*^2^ = 17%, P_Heterogeneity_ = 0.13 for p-acetamidophenylglucuronide; Table 1). Funnel plots appeared generally symmetrical for all the three metabolites, also suggesting no evidence for horizontal pleiotropy (Additional file 1: Fig. S1). Dihomo-linoleate (20:2n6) showed a negative association with intelligence (β_IVW_ = − 0.14; 95% CI − 0.25 to − 0.04), while the association between p-acetamidophenylglucuronide and intelligence was positive (β_IVW_ = 0.01; 95% CI 0.00 to 0.01). The causal association between 5-oxoproline and human intelligence is shown on Fig. 2, while the associations for dihomo-linoleate (20:2n6) and p-acetamidophenylglucuronide with intelligence are represented on Fig. 3. Notably, the very small effect size for p-acetamidophenylglucuronide on intelligence might limit its potential utility as a biomarker.

**Table 1:** Sensitivity analysis of causal associations between metabolites and intelligence

Metabolites	MR-Egger		Weighted median		MR-PRESSO		Pleiotropy test		Heterogeneity test	
	β (95% CI)	P-value	β (95% CI)	P-value	β (95% CI)	P-value	P_intercept_	P_Global test_	*I*^2^	P-value
Amino acid										
5-Oxoproline	0.36(0.18,0.55)	**0.0001**	0.31(0.18,0.45)	**6.29E-06**	0.24(0.12,0.35)	**0.0007**	0.09	0.06	25%	0.13
Indolelactate	− 0.11(− 0.27,0.06)	0.2135	− 0.11(− 0.23,0.02)	0.0872	− 0.09(− 0.17,− 0.02)	**0.0244**	0.85	0.76	0%	0.74
Carbohydrate										
Mannitol	-0.01(− 0.07,0.06)	0.9040	− 0.02(− 0.06,0.02)	0.2799	− 0.03(− 0.06,− 0.01)	**0.0138**	0.34	0.84	0%	0.82
Lipid										
2-Oleoylglycerophosphocholine	− 0.37(− 0.82,0.08)	0.1067	0.21(0.05,0.37)	**0.0117**	0.18(0.05,0.30)	**0.0141**	0.26	0.01	17%	0.26
2-Palmitoylglycerophosphocholine	0.06(− 0.11,0.24)	0.4860	0.11(− 0.01,0.23)	0.0682	0.14(0.04,0.25)	**0.0117**	0.26	< 0.01	45%	< 0.01
2-Stearoylglycerophosphocholine	− 0.01(− 0.39,0.38)	0.9734	0.11(− 0.01,0.22)	0.0554	0.11(0.03,0.20)	**0.0265**	0.54	0.43	4%	0.41
1-Oleoylglycerol (1-monoolein)	− 0.01(− 0.33,0.32)	0.9753	0.07(− 0.01,0.16)	0.0937	0.09(0.01,0.17)	0.0533	0.57	< 0.01	62%	< 0.01
Dihomo-linoleate (20:2n6)	− 0.33(− 0.66,− 0.01)	**0.0494**	− 0.17(− 0.32,− 0.02)	**0.0236**	− 0.14(− 0.25,− 0.04)	**0.0293**	0.24	0.17	0%	0.92
Docosapentaenoate (22:5n3)	0.18(− 0.03,0.40)	0.0993	− 0.16(− 0.28,− 0.03)	**0.0117**	− 0.16(− 0.29,− 0.03)	**0.0414**	< 0.01	< 0.01	71%	< 0.01
Linolenate (18:3n3 or 6)	− 0.37(− 0.73,− 0.01)	**0.0414**	− 0.19(− 0.38,− 0.01)	**0.0497**	− 0.20(− 0.38,− 0.03)	0.0814	0.30	0.08	2%	0.40
Acetylcarnitine	− 0.17(− 0.54,0.20)	0.3618	− 0.23(− 0.39,− 0.07)	**0.0053**	− 0.25(− 0.40,− 0.10)	**0.0044**	0.66	0.01	51%	< 0.01
Peptide										
Cyclo(leu-pro)	− 0.09(− 0.19,0.02)	0.1018	− 0.03(− 0.10,0.04)	0.4332	− 0.06(− 0.12,− 0.01)	**0.0438**	0.60	0.03	38%	0.07
Xenobiotics										
Stachydrine	0.10(− 0.05,0.25)	0.2068	0.04(− 0.02,0.10)	0.1482	0.06(0.02,0.11)	**0.0308**	0.65	0.41	11%	0.35
p-Acetamidophenylglucuronide	0.01(0.00,0.01)	**0.0075**	0.01(0.00,0.01)	**0.0060**	0.01(0.00,0.01)	**0.0454**	0.06	0.06	17%	0.13
Salicyluric glucuronide	1.00(0.98,1.02)	0.7825	0.99(0.97,1.01)	0.1379	0.99(0.98,0.99)	**0.0373**	0.05	0.48	0%	0.67
Hydroquinone sulfate	0.97(0.94,1.01)	0.204	0.97(0.94,1.01)	0.0865	0.98(0.96,0.99)	**0.0211**	0.96	0.69	0%	0.69

### Associations with other relevant outcomes

**Fig. 4:**
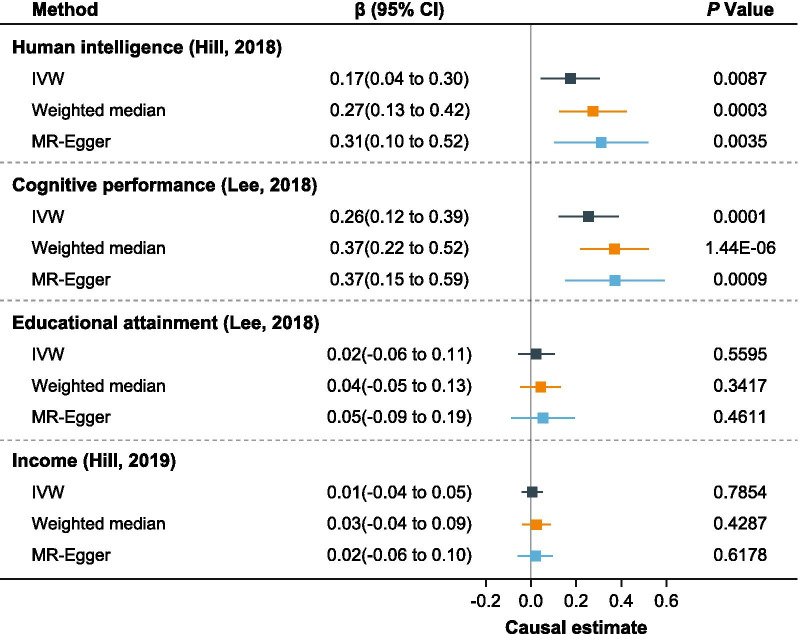
Mendelian randomization associations of 5-oxoproline on other intelligence-related outcomes from other data sources

We next repeated the main findings using summary statistics from other data sources. Figure 4 showed the results of causal effects of 5-oxoproline on human intelligence from another data source, cognitive performance, educational attainment, and income. The effect of genetically determined 5-oxoproline on intelligence (Replication) was similar (β = 0.17; 95% CI 0.04 to 0.30, P_IVW_ = 0.0087) to the result of initial MR estimates, and the causal associations were robust when different methods were performed (P_Weighted median_ = 0.0003, P_MR-Egger_ = 0.0035). The results also showed that 5-oxoproline was significantly associated with cognitive performance (P_IVW_ = 0.0001, P_Weighted median_ = 1.44 × 10^–6^, P_MR-Egger_ = 0.0009). However, no evidences for association were found between 5-oxoproline and educational attainment (P_IVW_ = 0.5595, P_Weighted median_ = 0.3417, P_MR-Egger_ = 0.4611), as well as income (P_IVW_ = 0.7854, P_Weighted median_ = 0.4287, P_MR-Egger_ = 0.6178). Besides, the effects of dihomo-linoleate (20:2n6) and p-acetamidophenylglucuronide on intelligence were also significant in the replication stage (Additional file 2: Fig. S2; Additional file 3: Fig. S3).

### Genetic basis for the causal associations

We further investigated the genetic variants that affected both metabolite levels and intelligence. Table 2 shows the 25 SNPs used as IV of 5-oxoproline. Among them, rs11986602 showed the most significant association with 5-oxoproline (β = − 0.0620; SE = 0.0029, P = 6.29 × 10^–104^). Notably, it also showed a strong association signal with intelligence (β = − 0.0196; SE = 0.0044, P = 9.53 × 10^–6^). Moreover, this SNP had the largest effect sizes on both 5-oxoproline and intelligence, suggesting that the related genetic locus might provide valuable information on the biological mechanisms of intelligence, and that 5-oxoproline might be an important functional intermediate to understand the biological process through which genetics affects intelligence. The IVs for dihomo-linoleate (20:2n6) and p-acetamidophenylglucuronide are shown in Additional file 4: Tables S3 and S4.

**Table 2:** Genetic predictors of 5-oxoproline and their association with Intelligence

SNP	Gene	CHR	A1	A2	5-oxoproline			Intelligence		
					Beta	SE	*P* value	Beta	SE	*P* value
rs11986602	EXOSC4	8	A	T	− 0.0620	0.0029	1.07E−104	− 0.0196	0.0044	9.53E−06
rs9987070	–	7	C	G	− 0.0280	0.0059	2.43E−06	− 0.0083	0.0071	0.2381
rs10890517	–	2	T	C	− 0.0197	0.0043	3.45E−06	− 0.0139	0.0069	0.0427
rs5764925	–	22	A	G	− 0.0160	0.0031	1.91E−07	− 0.0021	0.0050	0.6782
rs13159409	–	5	T	G	− 0.0148	0.0032	2.74E−06	− 0.0075	0.0053	0.1535
rs12294182	MICAL2	11	T	C	0.0140	0.0029	1.07E−06	0.0019	0.0041	0.6478
rs2068157	AACSP1	5	T	C	0.0137	0.0031	8.99E−06	− 0.0037	0.0048	0.4362
rs9964014	DLGAP1	18	T	C	− 0.0132	0.0025	1.80E−07	− 0.0060	0.0078	0.4388
rs11605366	–	11	T	C	− 0.0122	0.0027	7.55E−06	− 0.0111	0.0043	0.0094
rs12143589	–	1	A	G	0.0118	0.0023	3.38E−07	− 0.0024	0.0036	0.5034
rs13013224	LOC105369165	2	C	G	0.0113	0.0023	7.04E−07	− 0.0031	0.0035	0.3781
rs306676	–	13	A	G	0.0112	0.0024	2.48E−06	− 0.0027	0.0041	0.5047
rs9650466	MROH1	8	T	C	0.0110	0.0020	3.80E−08	0.0046	0.0028	0.0938
rs1001210	ATXN1	6	T	C	− 0.0106	0.0023	3.80E−−06	− 0.0051	0.0037	0.1678
rs17017431	TRAF5	1	A	T	0.0105	0.0023	3.80E−06	0.0022	0.0038	0.5628
rs10853533	SLC14A2	18	A	C	0.0103	0.0023	6.09E−06	− 0.0068	0.0043	0.1133
rs2115151	SPATA5	4	A	T	0.0103	0.0022	3.75E−06	0.0024	0.0041	0.5561
rs7015048	–	8	T	C	0.0100	0.0015	3.16E−11	− 0.0015	0.0028	0.5864
rs9460424	–	6	T	G	− 0.0097	0.0022	9.16E−06	− 0.0053	0.0035	0.1328
rs4646693	LRRK1	15	T	C	− 0.0090	0.0020	6.80E−06	− 0.0001	0.0054	0.9909
rs8092658	SLC14A2	18	A	C	− 0.0090	0.0020	6.80E−06	0.0001	0.0028	0.9828
rs1578743	–	10	A	C	0.0075	0.0016	1.70E−06	0.0026	0.0029	0.3664
rs7973508	–	12	A	G	− 0.0073	0.0016	5.40E−06	0.0007	0.0030	0.8245
rs12464424	–	2	T	C	− 0.0071	0.0016	7.47E−06	0.0055	0.0030	0.0651
rs12611788	GALNT14	2	T	C	− 0.0070	0.0015	5.51E−06	− 0.0062	0.0029	0.0301

### Metabolic pathway analysis

Table 3 shows the results of the metabolic pathway analysis. Based on the 16 known metabolites identified by the IVW method, we detected only one significant metabolic pathway associated with intelligence, namely *Alpha linolenic acid and linoleic acid metabolism* (P = 0.0062). Two metabolites identified by IVW, docosapentaenoate (n3 DPA; 22:5n3) and linolenate (18:3n3 or 6), are involved in *Alpha linolenic acid and linoleic acid metabolism* according to the SMPDB database. Importantly, many of the metabolites found by our analysis have not been assigned to any metabolic pathway currently recorded in the SMPDB or KEGG databases. Extensive further research will be needed to explore whether these metabolites are involved in biological processes relevant to differences in human intelligence.

**Table 3:** Results of metabolic pathway analysis

Metabolic pathway	Involved Metabolites	P value	Database
Alpha linolenic acid and linoleic acid metabolism	Docosapentaenoate (n3 DPA; 22:5n3); Linolenate (18:3n3 or 6)	0.0062	SMPDB
Alpha-linolenic acid metabolism	Linolenate (18:3n3 or 6)	0.0702	KEGG
Glutathione metabolism	5-Oxoproline	0.0912	KEGG
Beta oxidation of very long chain fatty acids	Acetylcarnitine	0.0989	SMPDB
Fructose and mannose metabolism	Mannitol	0.1140	KEGG
Oxidation of branched chain fatty acids	Acetylcarnitine	0.1622	SMPDB
Tryptophan metabolism	Indolelactate	0.1816	KEGG

## Discussion

We implemented a two-sample MR analysis to assess the causal relationships between genetically determined metabolites and human intelligence. Using genetic variants as IVs, we found that the genetically determined levels of 5-oxoproline were associated with better performance in human intelligence tests. This causal association was not affected by confounders such as educational attainment and household income, and was well replicated using samples from other data source. Our study also identified other metabolites and metabolic pathways involved in biological processes related to human intelligence, such as dihomo-linoleate (20:2n6) and p-acetamidophenylglucuronide. To the best of our knowledge, this is the first study combining information from genomics and metabolomics to assess the causal effects of metabolome traits on human intelligence.

5-Oxoproline, also known as pyroglutamic acid, is a cyclized derivative of l-glutamic acid that participates substantially in the glutamate and glutathione metabolism [34]. Disturbances in glutamate and glutathione metabolism can lead to a series of neurologic phenotypes, including developmental delay, ataxia, seizures, and intellectual disability [35]. Moreover, 5-oxoproline was also developed and sold as an over-the-counter “smart drug” for cognitive and memory improvement [36, 37]. However, it was also demonstrated that metabolic acidosis could be caused by excessive 5-oxoproline generation, with multiple adverse effects on many organ systems [38]. Our study found that elevated levels of 5-oxoproline were associated with a higher score in intelligence tests, supporting the potential usefulness of 5-oxoproline in improving intelligence-related performance. However, more work aimed at understanding the molecular mechanisms involved is needed to further clarify the role of this compound in human intelligence.

Genetic factors played a central role in our study of the causal relationship between metabolic traits and intelligence. The SNP rs11986602 (corresponding to the *EXOSC4* gene) was the most significantly associated to both 5-oxoproline levels and human intelligence. Although rarely discussed in the past literature, *EXOSC4* is known to be related to the protein kinase R (PKR)-like endoplasmic reticulum kinase (PERK, encoded by the *EIF2AK3* gene), which regulates gene expression [39]. A recent study reported that locally reduced PERK expression or activity could enhance neuronal excitability and improve memory and cognitive function in young mice [40]. Another study provided evidence that PERK is a key regulator of memory impairments and neurodegeneration in Alzheimer’s disease [41]. Thus, *EXOSC4* might be a causal risk gene participating in physiological processes important for human intelligence.

We further focused on the metabolic pathways that might be involved in the biological processes associated to human intelligence. The only identified metabolic pathway in our study was *Alpha linolenic acid and linoleic acid metabolism.* Alpha linolenic acid and linoleic acid are long-chain polyunsaturated fatty acids, which are essential nutrients in the development and functioning of the brain [42]. Many related compounds, such as alpha linolenic acid and docosahexaenoic acid, are involved in the rapid growth and development of the infant brain [43, 44]. Our study thus reinforced the importance of alpha linolenic acid and linoleic acid metabolism for human intelligence, providing valuable information for understanding the biological mechanisms related to human intelligence.

The current study has several strengths. First, we implemented a novel MR study design to assess the causal relationships between genetically determined metabolites and human intelligence. By using genetic variants as IVs, the MR approach prevents confounding, reverse causation, and various biases common in observational epidemiological studies. Second, our study provides, indirectly, a comprehensive assessment of the causal effects of metabolites assessed by non-targeted metabolomics on human intelligence. Third, by integrating genomics and metabolomics, our study provides novel insight into the biological mechanisms underlying differences in intelligence.

There are also several limitations that should be noted. First, the GWAS data for intelligence was determined adjusting for socioeconomic status, which was a heritable and correlated secondary trait to intelligence [29, 45]. The adjustment for socioeconomic status might cause bias in genetic associations with intelligence for some SNPs [46]. Second, our study could not avoid the bias of dynastic effect, which induced a correlation between the environment a child is raised in and their genetic inheritance and almost certainly violated the independence assumption of MR [47, 48]. Within family GWAS data was useful in avoiding the issue of dynastic effects. However, such data was not available at this stage. Third, our study failed to perform the bi-directional MR analysis which was useful in detecting false positive MR results arising from genetic correlation between traits. The reason was that many of the IVs for intelligence were missing in datasets of metabolites. Finally, the MR estimates from non-experimental date could not provide information towards molecular mechanism, further work should be done to determine the roles of metabolites or genetic variants in development of intelligence.

In summary, our study identified multiple metabolites that might have causal effects on human intelligence, among which 5-oxoproline presented significant association signals after Bonferroni correction. The association was shown to be robust by sensitivity analyses. Our study also highlighted that genetic factors (e.g. the *EXOSC4* gene) contributed substantially to the variation of metabolite levels and differences in human intelligence. Moreover, our findings suggest that alpha linolenic acid and linoleic acid metabolism might be involved in the biological processes underlying intelligence. Though further evidence from experimental data is needed, our study provides novel clues that would improve our understanding of the biological mechanisms related to human intelligence.

## Supplementary Information

**Additional file 1: Fig. S1**. Funnel plots for detecting potential pleiotropy.

**Additional file 2: Fig. S2.** Mendelian randomization associations of dihomo-linoleate(20:2n6) on other intelligence-related outcomes from other data sources.

**Additional file 3: Fig. S3.** Mendelian randomizationassociations of p-acetamidophenylglucuronide on other intelligence-relatedoutcomes from other data sources.

**Additional file 4:** **Table S1.** Information of the instrumental variables used for Mendelian randomization estimates. **Table S2.** Unknown metabolites identified by the Mendelian randomization estimates.** Table S3.** Genetic predictors for dihomo-linoleate (20:2n6) and their association with Intelligence. **Table S4.** Genetic predictors for p-acetamidophenylglucuronide and their association with Intelligence.

## Data Availability

Full summary statistics for the 486 metabolites are publicly available at the Metabolomics GWAS Server (http://metabolomics.helmholtz-muenchen.de/gwas/). GWAS summary statistics for intelligence are download from http://ctg.cncr.nl/software/summary_statistics.

## Abbreviations

GDM: Genetically determined metabotype
MR: Mendelian randomization
IVW: Inverse-variance weighted
IV: Instrumental variable
GWAS: Genome-wide association study
SNP: Single nucleotide polymorphism
InSIDE: Instrument strength independent of direct effect
SMPDB: Small Molecule Pathway Database
KEGG: Kyoto Encyclopedia of Genes and Genomes
