# Supplementary material for: Causal relationships between genetically determined metabolites and human intelligence: a Mendelian randomization study
Source: Mol Brain. 2021 Feb 9;14:29. doi: 10.1186/s13041-021-00743-4 (PMC7871559; doi:10.1186/s13041-021-00743-4)

**a. Funnel plot for 5-oxoproline**

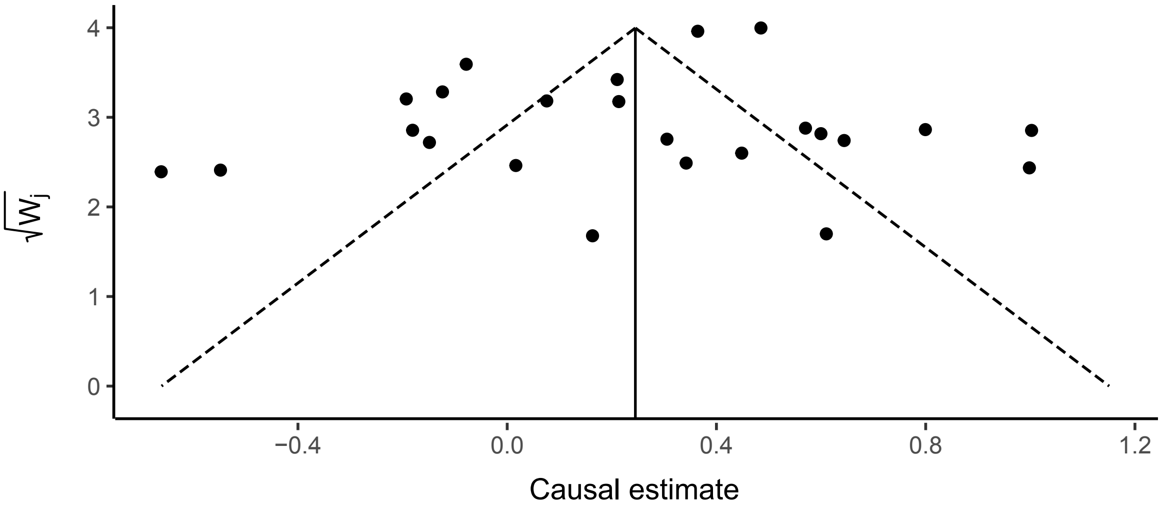


**b. Funnel plot for dihomo-linoleate (20:2n6)**

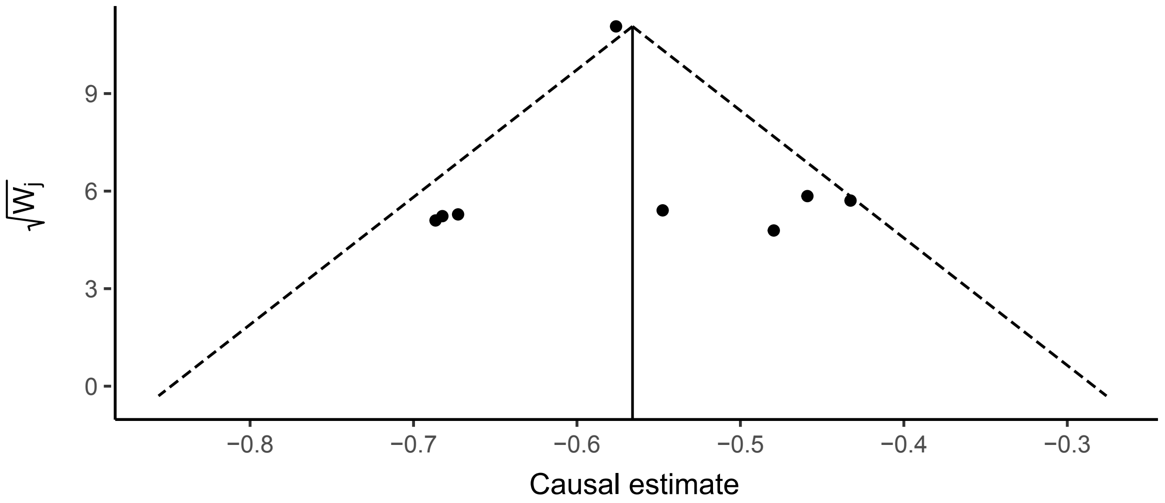


**c. Funnel plot for p-acetamidophenylglucuronide**

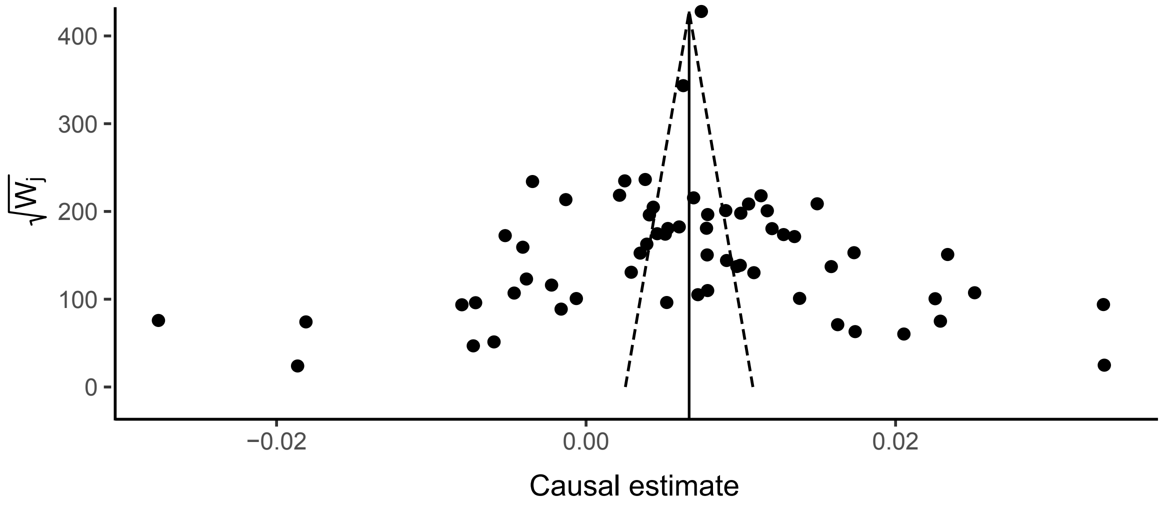

Supplement: Supplementary file 1 — Additional file 1: Fig. S1. Funnel plots for detecting potential pleiotropy. [file 13041_2021_743_MOESM1_ESM.pdf]
