# Supplementary figures and images for: Causal relationships between genetically determined metabolites and human intelligence: a Mendelian randomization study
Source: Mol Brain. 2021 Feb 9;14:29. doi: 10.1186/s13041-021-00743-4 (PMC7871559; doi:10.1186/s13041-021-00743-4)

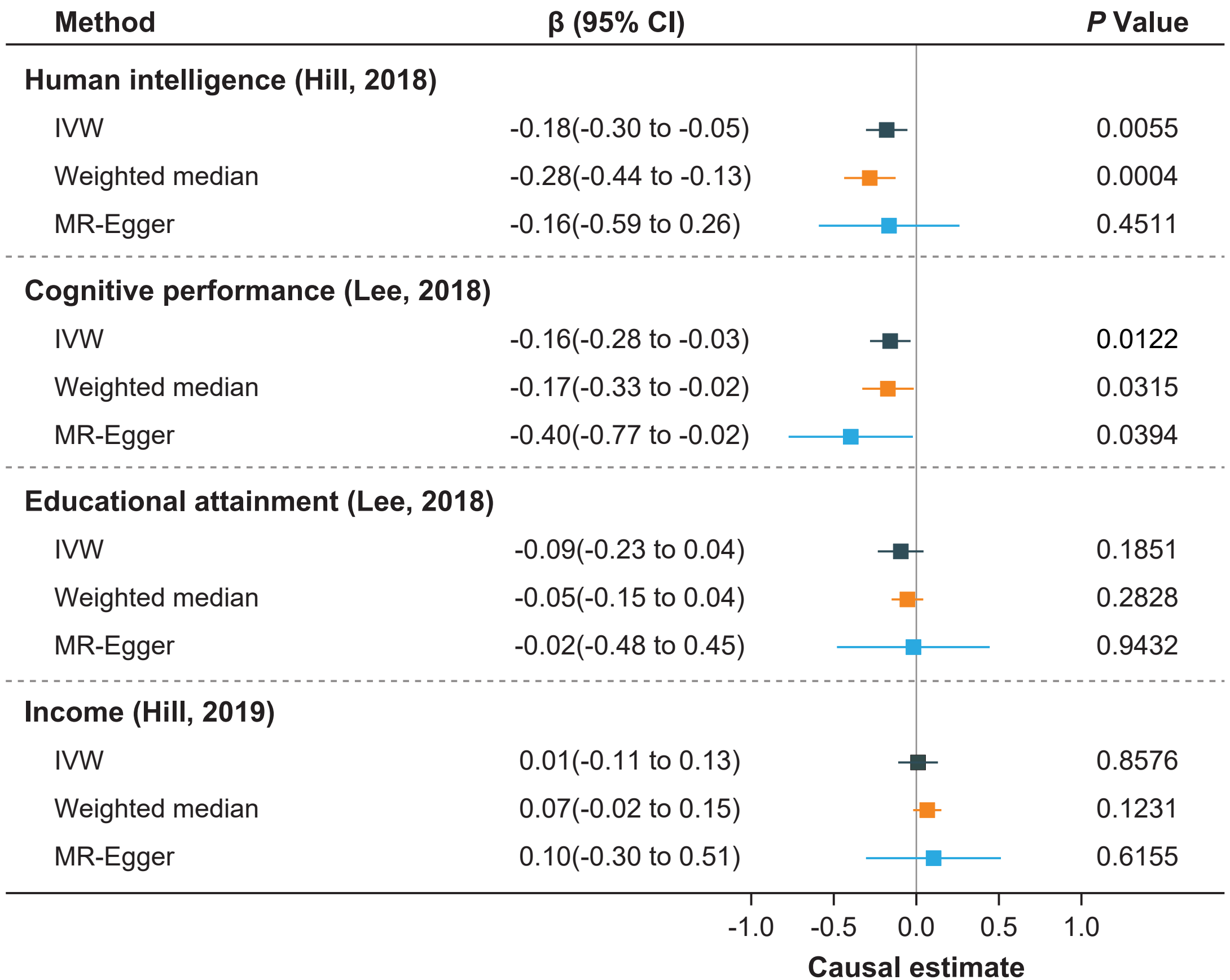

Supplement: Supplementary file 2 — Additional file 2: Fig. S2. Mendelian randomization associations of dihomo-linoleate(20:2n6) on other intelligence-related outcomes from other data sources. [file 13041_2021_743_MOESM2_ESM.pdf]

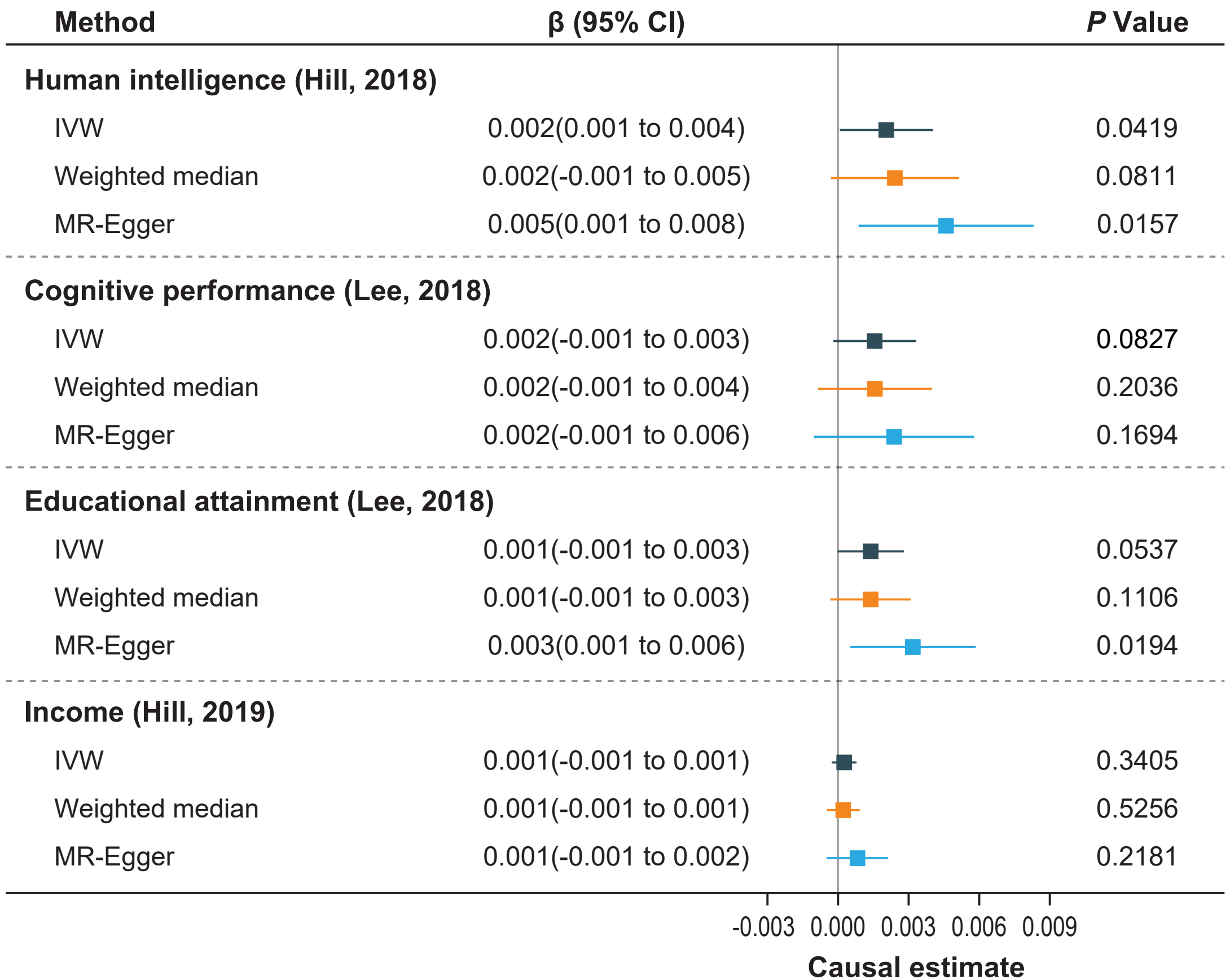

Supplement: Supplementary file 3 — Additional file 3: Fig. S3. Mendelian randomizationassociations of p-acetamidophenylglucuronide on other intelligence-relatedoutcomes from other data sources. [file 13041_2021_743_MOESM3_ESM.pdf]
